# P-508. Factors Influencing Feasibility of Studying Pregnancy and Infant Outcomes After Antenatal Lyme Disease

**DOI:** 10.1093/ofid/ofaf695.723

**Published:** 2026-01-11

**Authors:** Meagan E Williams, Roberta L DeBiasi, David Schwartz, Sarah B Mulkey

**Affiliations:** Children's National Hospital, Washington, DC; Children's National Hospital/The George Washington University School of Medicine and Health Sciences, Washington, District of Columbia; Perinatal Pathology Consulting, Atlanta, Georgia; Children's National Hospital/ George Washington University School of Medicine and Health Sciences, Washington, DC

## Abstract

**Background:**

Effects of antenatal Lyme disease (LD) exposure on the pregnant patient, placenta, and fetus and subsequent child outcomes are not well known. To date, there have been no obstetrical pathology and long-term neurodevelopmental follow-up studies of infants exposed to LD during pregnancy. A first-of-its-kind pilot study (NCT06026969) is being conducted at Children’s National Hospital in Washington, DC.

**Methods:**

This study aims to assess the feasibility of recruiting pregnant patients with LD and following their pregnancy outcomes and children’s neurodevelopment through age 18 months. Pregnant adults in the U.S. or Canada can participate if they have met CDC criteria for acute LD during their current pregnancy or post-treatment Lyme disease syndrome (PTLDS) within 5 years.

**Results:**

As of May 2025, 9 participants (4 with acute LD, 5 with PTLDS) have enrolled in the study. Successful recruitment methods have included posting in LD-focused social media groups and partnering with trusted stakeholders (doctors, nonprofit organizations) to share details about the study. All enrolled participants have learned about the study online. Fifty additional people have completed an interest screener; reasons for non-enrollment include not meeting CDC criteria for LD/PTLDS (n=15) or diagnosis outside of the eligibility window (n=7), not being pregnant at the time of eligibility assessment (n=5), them declining participation (n=6), or eligibility assessment in progress (n=17).

Eight participants have given birth to date; 100% have completed neurodevelopmental follow-up and a qualitative interview about their experience with LD/PTLDS during pregnancy. Four have completed prenatal and/or postnatal imaging. Placentas have been successfully processed by all birth hospitals in preparation for further analysis.

**Conclusion:**

While recruitment/enrollment are complex, there is robust interest from the pregnant LD/PTLDS patient population in contributing to research. Reducing financial barriers (e.g., travel costs), early communication with birth hospitals about placenta collection, partnering with trusted stakeholders, and using targeted marketing strategies have contributed to success in enrolling participants at this infrequent intersection of LD and pregnancy.

**Disclosures:**

Roberta L. DeBiasi, MD, MS, Pfizer: Grant/Research Support Sarah B. Mulkey, MD, PhD, Pfizer: Advisor/Consultant

